# Variation among cardiovascular risk calculators in relative risk increases with identical risk factor increases

**DOI:** 10.1186/s13104-015-1401-8

**Published:** 2015-09-07

**Authors:** G. Michael Allan, Faeze Nouri, Christina Korownyk, Michael R. Kolber, Ben Vandermeer, James McCormack

**Affiliations:** Evidence-Based Medicine, Department of Family Medicine-Research Program, University of Alberta, 6-10 University Terrace, Edmonton, AB T6G 2T4 Canada; Alberta Research Centre for Health Evidence, University of Alberta, Edmonton, AB Canada; Faculty of Pharmaceutical Sciences, University of British Columbia, Vancouver, BC Canada

**Keywords:** Cardiovascular risk calculator, Cardiovascular risk prediction, Relative risk, Relative risk increase, Risk factor

## Abstract

**Background:**

Risk estimates for the same patient can vary substantially among cardiovascular risk calculators and the reasons are not fully explained. We compared the relative risk increases for consistent risk factors changes across different cardiovascular risk calculators.

**Methods:**

Five clinicians independently selected 16 calculators providing absolute risk estimations. Hypothetical patients were generated using a combination of seven risk factors [age, gender, smoking, blood pressure, high-density lipoprotein (HDL), total cholesterol and diabetes] dichotomized to high and low risk, generating 2^7^ patients (128 total). Relative risk increases due to specific risk factors were determined and compared.

**Results:**

The 16 selected calculators were from six countries, used 5- and 10-year predictions, and estimated CVD or coronary heart disease risk. Across the different calculators for non-diabetic patients, changing age from 50 to 70 produced average relative risk increases from 82 to 395 %, gender (female to male) 35–225 %, smoking status 31–118 %, systolic blood pressure (120–160 mmHg) 16–124 %, total cholesterol (4–7 mmol/L) 51–302 % and HDL (1.3–0.8 mmol/L) 27–133 %. Similar results were found among diabetic patients. Some calculators appeared to have consistently higher relative risk increases over multiple risk factors.

**Conclusions:**

Cardiovascular risk calculators weigh the same risk factors differently. For each risk factor, the relative risk increase from the calculator with the highest increase was generally three to eight times greater than the relative risk increase from the calculator with lowest increase. This likely contributes to some of the inconsistency in risk calculator estimation. It also limits the use of risk calculators in estimating the benefits of therapy.

**Electronic supplementary material:**

The online version of this article (doi:10.1186/s13104-015-1401-8) contains supplementary material, which is available to authorized users.

## Background

Guidelines frequently encourage clinicians to use cardiovascular disease (CVD) risk calculators to estimate a patient’s cardiovascular risk. The information is often used to classify patients into different risk categories to guide treatment decisions. Alternatively, calculators can provide absolute values to explain a patient’s estimated risk and discuss the benefits of differing therapies.

Although up to 74 % of primary care physicians who are specifically interested in cardiac disease may regularly use CVD risk calculators [1], most studies show only 22–48 % physicians regularly use risk calculators [2–4]. Some of the diverse reasons why calculators have not been universally adopted include lack of time, a feeling that the information is not helpful, a sense of oversimplification with risk tools, and an ability to predict risk subjectively [2–4].

The inconsistency among CVD risk calculators [5–8] presents another possible limitation to their adoption and application. A review of 25 risk calculators found 33 % of the time different calculators assigned the same patient to a different risk category [8]. For individual patients, the highest calculated absolute risk was, on average, five times higher than the lowest calculated absolute risk [8]. Focusing on diabetic or non-diabetic patients, cardiovascular or coronary heart disease outcomes, and/or 5- or 10-year time horizons made little difference in risk calculation agreement [8]. Only by focusing on CVD outcomes with a 10-year time horizon from Framingham-derived calculators did agreement approach 90 % [8].

Although inconsistency is common among risk calculator estimations, the causes of the broad variation in absolute risk and frequent disagreement in risk category assignment has not yet been described. As identified above, eliminating most of the variables appeared to improve agreement but did not fully explain the reasons for disagreement. Our objective in this study was to determine how different risk calculators weigh individual cardiovascular risk factors (e.g. smoking).

## Methods

This is a sub-study of risk factor increases from our initial study of CVD risk calculator agreement [8]. Selection of calculators and generation of hypothetical patients has been explained previously [8] but are reviewed below.

### Calculator selection

**Fig. 1:**
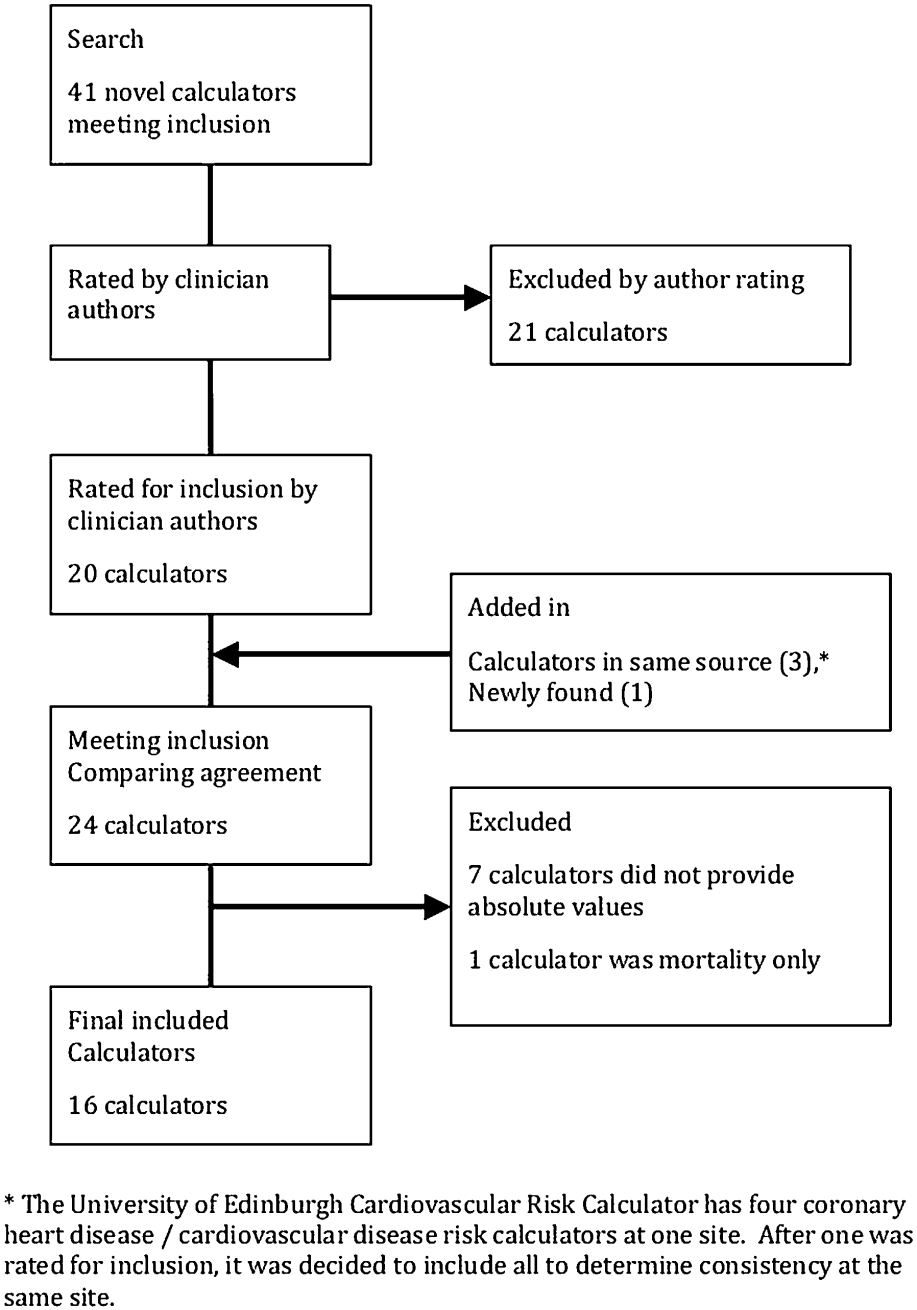
Flow of identification and selection of cardiovascular risk calculators for inclusion

We searched for and then independently selected a broad range of CVD and coronary heart disease (CHD) risk calculators. Our goal was to identify representative sample of calculators from different countries, were or were not associated with guidelines, used different data sources (Framingham and others), used different formats (internet, paper and pencil, other) and calculated different outcomes durations (5 or 10 years). Originally, 20 calculators were selected and 4 more were added to enhance diversity. In this sub-study, absolute risk estimates are needed to compare the relative risk increases that result from risk factor changes. Therefore, we excluded seven calculators that did not provide absolute numbers. We also excluded the SCORE calculator as it only provided CVD mortality risk. Calculator inclusion and exclusion flow are presented in Fig. 1.

### Patient variables

Seven risk factors common to all included calculators were age, gender, smoking status, diabetes, systolic BP, total cholesterol and HDL (or the total cholesterol/HDL ratio). Using a specific increase for each risk factor facilitated comparison across different calculators. We assigned two values for each risk factor: age 70 or 50; gender male or female; current smoking status yes or no; systolic BP 160 or 120 mmHg; total cholesterol 7 or 4 mmol/L; HDL 0.8 or 1.3 mmol/L. The two values for each seven risk factors created 2^7^ or 128 distinct patients.

Two authors independently completed the risk assessment for all 128 hypothetical patients on each calculator. Ethics approval was not required for this study (as patients were hypothetical).

### Analysis

#### Calculation of relative risk increase

To analyze the relative risk increase associated with each risk factor from each calculator, we performed individual risk factor analysis. For individual risk factor analysis (i.e., BP), we started with the lower risk option for that risk factor (120 mmHg) and then varied other risk factors (age, gender, smoking status, total cholesterol, HDL, and diabetes) between high and low risk values to create 64 unique patients. For each patient, we increased the individual risk factor to the high-risk option (i.e. 160 mmHg) and recalculated the risks. This created pairs of identical patients with the same risk factors except for the individual risk factor analyzed, which was low-risk and high-risk for each patient in the pair. We then subtracted the lower-risk estimate from the higher-risk estimate, and divided the risk difference by the low-risk estimate. This created the patient’s relative risk increase for the specific individual risk factor.

As an example, one patient pair in the BP risk factor analysis started with a 50 year old, male, smoker, non-diabetic, with a 7 mmol/L total cholesterol, and a 0.8 mmol/L HDL. The low-risk patient had a 120 mmHg systolic BP while the high-risk was 160 mmHg. Edinburgh ASSIGN risk calculator estimated the low-risk patient’s risk (with the 120 mmHg BP) as 16.6 % and the high-risk patient’s risk (with the 160 mmHg BP) as 25.3 %. The calculated relative risk increase would be (25.3–16.6 %)/16.6 % = 52.4 %.

#### Mean relative risk increase

The 128 patients provided 64 patient pairs in each individual risk factor analysis. As some calculators do not estimate risk in diabetic patients, diabetic and non-diabetic patients were analyzed and presented separately. This halved the numbers again, leaving 32 diabetic and 32 non-diabetic patient pairs. Therefore, each risk factor analysis had 32 relative risk increases for each calculator (among diabetics or non-diabetics). For each risk factor, we calculated the mean and standard deviation of the relative risk increase of each calculator. An example of the full formula is below.
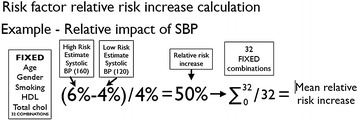


For each risk factor, we ranked calculators from lowest to highest average relative risk increase. We also performed a sub-group analysis when permitted by the number of calculators per sub-group.

## Results

Characteristics of the 16 calculators, 10 of which included diabetics, are summarized in Table 1 [9–21].

**Table 1:** Characteristics of included risk calculators

Risk calculator	Composite outcome	Time frame	Include diabetes	Design	Country	Cohort data used
Edinburgh (Framingham CVD) [9]	CVD	10 (can choose)	Yes	Web	UK	Framingham
Edinburgh (ASSIGN) [9]	CVD	10	Yes	Web	UK	ASSIGN
Primary CVD risk calculator [10]	CVD	10	Yes	Web	UK	Framingham
Framingham Heart Study [11]	CVD	10	Yes	Web	USA	Framingham
QRISK2-2011 [12]	CVD	10	Yes	Web	UK	GP data in UK
Progetto Cuore [13]	CVD	10	Yes	Web	Italy	Longitudinal Italian Studies
JBS Assessor [14]	CVD	10	No	Download	UK	Framingham
Edinburgh (BNF) [9]	CVD	10	No	Web	UK	BNF
Reynolds Risk Score [15]	CVD	10	No	Web	USA	Reynolds
Australian absolute CVD risk [16]	CVD	5	No (ranges for diabetes)	Web	Australia	National Vascular Disease Prevention Alliance
New Zealand know your numbers [17]	CVD	5	Yes	Web	New Zealand	Framingham
UKPDS risk engine [18]	CHD	10	Yes	Download	UK	UKPDS
i-phone STAT ATPIII lipid management [19]	CHD	10	No	i-phone	USA	Framingham
Edinburgh (Framingham CHD) [9]	CHD	10	Yes	Web	UK	Framingham
PROCAM health check [20]	CHD	10	Yes	Web	Germany	German Data
National cholesterol Education Program [21]	CHD (MI)	10	No	Web	USA	Framingham

### Comparison of risk increase due to specific risk factors

For non-diabetic patients, the mean relative risk increases for each risk factor and calculator are summarized on Table 2. The mean relative risk increase varied considerably across calculators. For example, changing age from 50 to 70 years, the average relative risk increase ranged from 82 % for Edinburgh (Framingham, CHD) to 395 % for PROCAM (Health Check), a 4.8 times difference. The ratios of highest average relative increase divided by lowest for the other risk factors was 6.4 for gender, 3.8 for smoking status, 7.8 systolic BP, 5.9 total cholesterol and 4.9 for HDL.

**Table 2:** Average relative risk increase due to specific risk factor for each included risk calculator among non-diabetic patients (relative risk increase ± standard deviation)

Risk calculator	Age (50–70) (%)	Gender (female to male) (%)	Smoking (non-smoker to smoker) (%)	Systolic BP (120–160 mmHg) (%)	Total chol (4–7 mmol/L) (%)	HDL (1.3–0.8 mmol/L) (%)
Edinburgh (Framingham CVD)	112 ± 35	44 ± 12	70 ± 24	73 ± 25	51 ± 17	42 ± 14
Edinburgh (ASSIGN)	222 ± 53	35 ± 28	37 ± 8	49 ± 6	60 ± 21	27 ± 3
Primary CVD risk calculator	103 ± 31	56 ± 18	59 ± 15	69 ± 17	66 ± 20	56 ± 19
Framingham Heart Study	121 ± 23	91 ± 33	67 ± 11	83 ± 26	78 ± 13	41 ± 7
QRISK2-2011	335 ± 91	53 ± 20	57 ± 20	30 ± 17	51 ± 15	43 ± 14
Progetto CUORE	313 ± 30	141 ± 57	87 ± 27	75 ± 13	66 ± 26	29 ± 4
JBS Assessor	110 ± 35	48 ± 16	59 ± 15	43 ± 13	63 ± 19	54 ± 18
Edinburgh (BNF)	105 ± 31	56 ± 18	59 ± 13	71 ± 19	68 ± 22	56 ± 18
Reynolds Risk Score	344 ± 96	119 ± 82	83 ± 50	124 ± 44	89 ± 42	60 ± 35
Australian absolute CVD risk	153 ± 50	61 ± 20	95 ± 38	98 ± 37	65 ± 20	55 ± 19
New Zealand Know Your Numbers	154 ± 49	60 ± 19	95 ± 37	99 ± 37	60 ± 22	50 ± 21
UKPDS Risk Engine	182 ± 16	79 ± 8	31 ± 3	16 ± 2	77 ± 8	64 ± 6
i-phone STAT ATPIII lipid management	210 ± 228	225 ± 140	116 ± 110	72 ± 32	154 ± 124	61 ± 14
Edinburgh (Framingham CHD)	82 ± 32	64 ± 26	55 ± 16	51 ± 5	88 ± 26	73 ± 22
PROCAM Health Check	395 ± 204	114 ± 82	118 ± 27	35 ± 8	302 ± 67	133 ± 30
National Cholesterol Education Program	198 ± 203	201 ± 124	110 ± 125	69 ± 49	141 ± 129	61 ± 33
Overall average	196 ± 140	91 ± 80	75 ± 54	66 ± 37	92 ± 80	57 ± 30

For diabetic patients, the mean relative risk increases for each risk factor and calculator are summarized on Table 3. The ratios of highest average relative increase divided by lowest was 4.9 for age, 18.2 for gender, 3.5 for smoking status, 4.9 systolic BP, 8.1 total cholesterol, 4.9 for HDL and 3.4 diabetic status.

**Table 3:** Average relative risk increase due to specific risk factor for each included risk calculator among diabetic patients (relative risk increase ± standard deviation)

Risk calculator	Age (50–70) (%)	Gender (female to male) (%)	Smoking (non-smoker to smoker) (%)	Systolic BP (120–160 mmHg) (%)	Total chol (4–7 mmol/L) (%)	HDL (1.3–0.8 mmol/L) (%)	Diabetes (yes or no) (%)
Edinburgh (Framingham CVD)	79 ± 28	11 ± 6	50 ± 18	52 ± 19	37 ± 13	31 ± 11	62 ± 33
Edinburgh (ASSIGN)	172 ± 47	15 ± 21	31 ± 10	40 ± 10	49 ± 20	23 ± 6	111 ± 28
Primary CVD risk calculator	96 ± 30	9 ± 10	52 ± 11	66 ± 16	51 ± 18	44 ± 16	60 ± 31
Framingham Heart Study	104 ± 24	62 ± 27	59 ± 14	73 ± 27	68 ± 17	35 ± 9	70 ± 18
QRISK2-2011	197 ± 65	37 ± 19	54 ± 20	28 ± 17	47 ± 14	40 ± 13	84 ± 37
Progetto CUORE	297 ± 35	164 ± 63	84 ± 28	72 ± 14	63 ± 25	28 ± 4	46 ± 8
New Zealand know your numbers	74 ± 13	11 ± 6	48 ± 7	49 ± 7	31 ± 6	25 ± 6	158 ± 123
UKPDS risk engine	167 ± 22	73 ± 11	29 ± 4	15 ± 2	72 ± 11	60 ± 9	50 ± 7
Edinburgh (Framingham CHD)	67 ± 30	17 ± 16	44 ± 13	41 ± 12	70 ± 20	58 ± 17	53 ± 31
PROCAM health check	327 ± 196	76 ± 65	101 ± 38	31 ± 11	251 ± 93	113 ± 42	88 ± 32
Overall average	158 ± 113	48 ± 56	55 ± 28	47 ± 24	74 ± 69	46 ± 30	78 ± 57

### Ranking of calculators by mean relative risk increase

For each risk factor, ranking of calculators based on mean relative risk increases from lowest to highest among non-diabetic and diabetic patients is provided in Table 4. Among the 16 non-diabetic calculators, Edinburgh (Framingham CVD), Edinburgh (ASSIGN), QRISK2-2011 and JBS Assessor had the lowest relative risk increase (ranked four or less) for three or four of the six risk factors. Reynolds Risk Score, iPhone ATPIII lipid management, PROCAM (Health Check), and National Cholesterol Education Program had the highest relative risk increases (ranked 13 or more) for three or four of the six risk factors.

**Table 4:** Ranking of each calculator by the average relative risk increase for each risk factor

Risk calculators	Age (50–70)	Gender (female to male)	Smoking (non-smoker to smoker)	Systolic BP (120–160 mmHg)	Total chol (4–7 mmol/L)	HDL (1.3 to 0.8 mmol/L)	Diabetes (non-diabetic to diabetic)
Non-diabetic patients							
Edinburgh (Framingham CVD)	5	2	9	11	1	4	
Edinburgh (ASSIGN)	12	1	2	5	4	1	
Primary CVD risk calculator	2	5	6	8	7	10	
Framingham Heart Study (CVD)	6	11	8	13	11	3	
QRISK2-2011	14	4	4	2	2	5	
Progetto CUORE	13	14	11	12	8	2	
JBS Assessor	4	3	7	4	5	7	
Edinburgh (BNF)	3	6	5	9	9	9	
Reynolds Risk Score	15	13	10	16	13	11	
NZ know your numbers	8	7	13	15	3	6	
Australian absolute CVD risk	7	8	12	14	6	8	
UKPDS	9	10	1	1	10	14	
iPhone ATP III lipid management	11	16	15	10	15	13	
Edinburgh (Framingham CHD)	1	9	3	6	12	15	
PROCAM (Health Check)	16	12	16	3	16	16	
National Cholesterol Education Program	10	15	14	7	14	12	
Diabetic patients							
Edinburgh (Framingham CVD)	3	2	5	7	2	4	5
Edinburgh (ASSIGN)	7	4	2	4	4	1	9
Primary CVD risk calculator	4	1	6	8	5	7	4
Framingham Heart Study (CVD)	5	7	8	10	7	5	6
QRISK2-2011	8	6	7	2	3	6	7
Progetto CUORE	9	10	9	9	6	3	1
NZ know your numbers	2	3	4	6	1	2	10
UKPDS	6	8	1	1	9	9	2
Edinburgh (Framingham CHD)	1	5	3	5	8	8	3
PROCAM (Health Check)	10	9	10	3	10	10	8

Among the 10 diabetic calculators, Edinburgh (Framingham CVD), NZ Know Your Numbers, UKPDS, and Edinburgh (Framingham CHD) had the lowest relative risk increase (ranked three or less out of 10 calculators) for three or four of the seven risk factors. Progetto CUORE, UKPDS and PROCAM (Health Check) had the highest relative risk increases (ranked eight or more out of ten calculators) for three or four of the seven risk factors.

### Sub-group comparison

Additional file 1: Table S1 provides the subgroup comparisons of the relative risk increase among non-diabetic calculators. Only two five-year calculators were identified and, therefore, were excluded. We did not complete subgroup analysis on diabetic calculators, as the number of calculators was too small.

Among 16 calculators for non-diabetic patients, there were 14 10-year calculators, 5 for CHD and 9 for CVD. The average relative risk increases were higher for CHD compared to CVD for all risk factors except BP. Additionally, the range of relative risk increases across the sample of calculators was higher for CHD compared to CVD for all risk factors except BP.

Among the 16 non-diabetic calculators, half were derived from the Framingham database and the other half from eight different databases. We excluded the 5-year risk calculators and the CHD calculators to focus the comparison on the four Framingham-derived 10-year CVD calculators versus the five non-Framingham-derived 10-year CVD calculators. The average relative risk increases for Framingham- and non-Framingham-derived calculators were remarkably similar, except for age. The range of relative risk increases was higher for non-Framingham-derived calculators compared to Framingham-derived calculators. Therefore, although the pooled relative risk increases are similar, there is more variability between non-Framingham-derived calculators.

### Sample patient

**Fig. 2:**
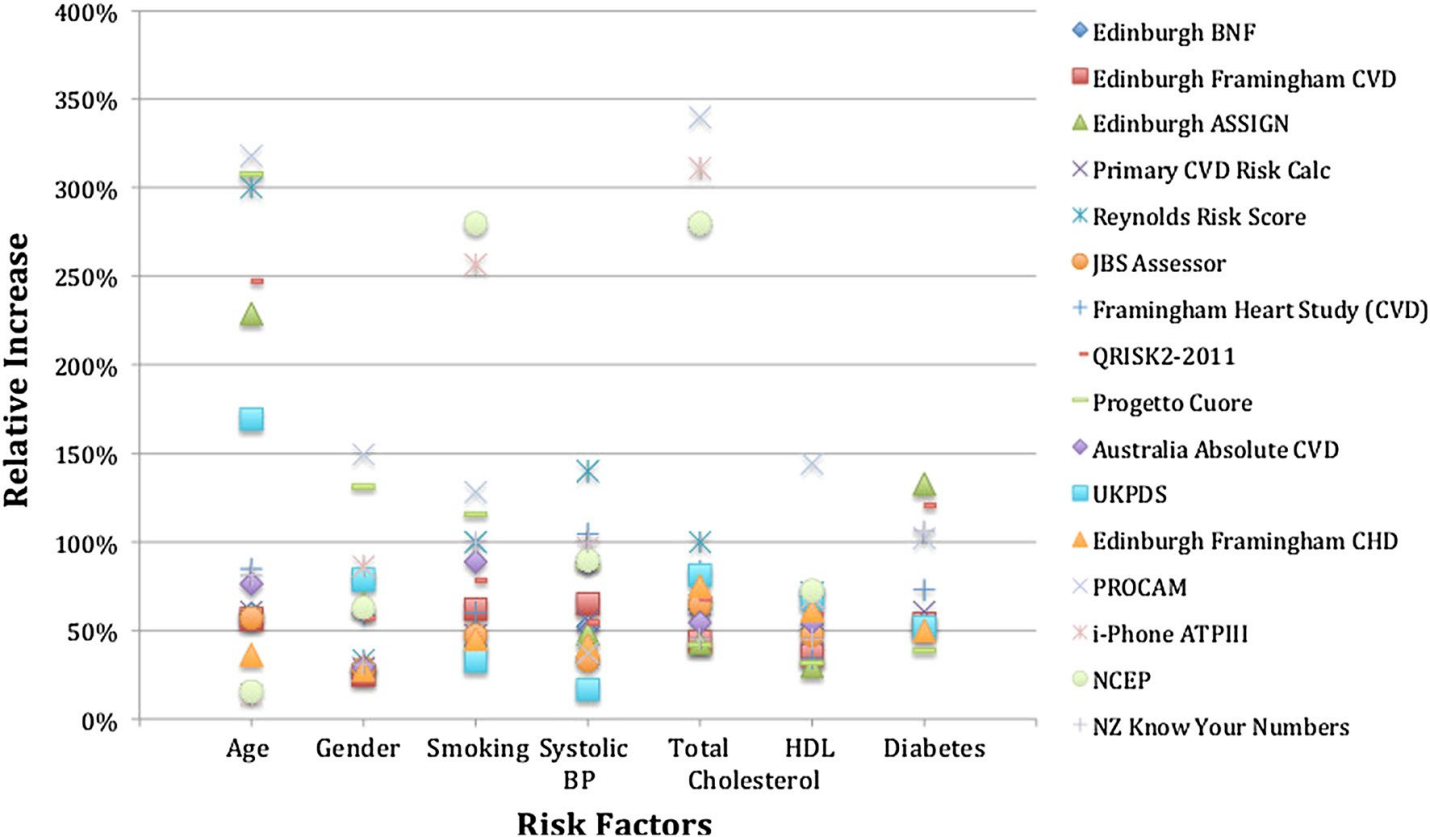
Sample of relative risk increases for each risk factor for a 50-year-old, female, smoker, 160 mmHg systolic blood pressure, 7 mmol/L total cholesterol, 0.8 mmol/L HDL, non-diabetic

Figure 2 illustrates the relative risk increases for each calculator when individual risk factors are changed for a sample patient. The sample patient is a 50-year-old, female, non-diabetic, smoker, with 160 mmHg systolic BP, 7 mmol/L total cholesterol, and 0.8 mmol/L HDL. For example, changing total cholesterol from 4 to 7 mmol/L, the relative risk increase for Progetto CUORE was 42 and 340 % for PROCAM.

## Discussion

The mean relative risk increase for specific risk factors varied considerably among the CVD risk calculators. The highest average relative risk increase from a calculator was generally three to eight times greater than the calculator with the lowest average. For example, among non-diabetic risk calculators, an increase in total cholesterol from 4 to 7 mmol/L resulted in an average relative risk of 302 % using the PROCAM Health Check Calculator compared to 51 % using the Edinburgh (Framingham CVD) calculator.

While there was a lot of variation among the overall group of calculators assessed, some calculators had remarkably similar results. For example, among non-diabetic calculators, the average relative risk increases for JBS Assessor and Edinburgh (BNF) were frequently within 8 % for 5 of 6 risk factors (the other 28 %). Alternatively, considering the relative risk increases associated with smoking among non-diabetics, the lowest average relative risk increase was 31 % while the highest was 118 %, a spread of 87 %. However, 11 of the 16 calculators had an average relative risk increase from 55 to 95 %, a difference of only 40 %. Similar results were found for gender that had a spread of 190 % (35–225 %), although 9 of 16 calculators were within a 29 % spread and 13 of 16 were within an 84 % spread.

Past research has shown frequent disagreement among risk calculators [5–8]. In our previous work, the disagreement in risk categorization occurred 33 % of the time for paired risk calculator comparisons [8]. As well, the absolute risk estimation varied considerably among different calculators, with the highest estimate five times greater than the lowest estimate for the same patient. In the previous study, subgroup testing found that limiting analysis to 5- or 10-year outcomes or CVD or CHD outcomes, did not meaningfully improve agreement. Agreement only improved by limiting analysis to 10-year CVD outcomes all derived from Framingham [8].

This is the first study to explore the reasons for differences in risk estimation. It is possible that poor agreement could have arisen, in part, from different baseline risk. However, as our original study showed, the lowest risk patient had remarkably similar risk estimation (≤4 %) across 14 different calculators. Alternatively, the weighting of risk factors among calculators could have been variable from patient to patient without any distinct pattern. However, we found large variation in how calculators weight risk factors, with some calculators consistently providing more or less weight for a certain risk factors.

Why is there a difference in relative risk increase among the calculators? Compared to calculators using a CVD endpoint, the relative risk increase for all risk factors for CHD calculators was higher, except systolic BP. As CVD includes stroke (and systolic BP is an important risk factor for stroke), the higher relative risk increases for systolic BP in CVD as compared to CHD calculators makes sense. Compare this to total cholesterol which had an average relative risk increase of 152 % in CHD calculators versus 56 % in CVD calculators. Thus, some of the variability in relative risk increases result from the type of outcomes assessed.

The database used to derive the calculator appears to play some role in the variability of the relative risk increase. Calculators derived from non-Framingham databases varied more broadly than those derived from Framingham. This corresponds to the results of our first study showing that agreement was better among Framingham-derived calculators than among non-Framingham-derived calculators [8]. As different populations have different risks and susceptibility to CVD, the variability with calculators derived from other databases could be anticipated. The Framingham-derived calculators must modify their model to better predict outcomes in different populations [22].

The variability in relative risk increases raises important concerns around the use of calculators to assess the benefits of therapeutic interventions that target specific risk factors. Although it may seem reasonable to estimate potential therapy benefits simply by changing the risk factor in the calculator, there are a number of problems with this approach. Clinicians cannot assume that medications taking a systolic BP from 160 to 140 mmHg will yield the clinical benefits similar to the risk difference between 160 and 140 mmHg. Additionally, we know that some interventions that modify risk factors (atenolol [23], torcetrapib [24], rosiglitazone [25] to name a few) do not reduce CVD. Another potential failing in this approach, identified in this study, is the different weighting that calculators appear to place on some risk factors. A change in systolic BP from 120 to 160 mmHg increases the average patient’s risk by 16 % (using the UKPDS engine) to 124 % (Reynolds Risk Score), a greater than seven-fold difference. Some studies try to estimate the absolute cardiovascular benefits from interventions designed to modify risk factors (e.g. naturopathic medicine for CVD risk [26]). By using changes in CVD estimations in this way, careful selection of risk calculator will result in dramatic difference in the calculated risks, and therefore perceived benefit of the intervention. For example, if the Reynolds Risk Score was used to estimate risk associated with BP changes, the estimated absolute cardiovascular risk benefit would be much greater than if the UKPDS calculator was used. Researchers, journal editors and reviews should recognize that calculator choice in these studies could easily change the results in both a statistical and clinically significant way.

### Limitations

Many of the limitations of this sub-study mirror those of our original study [8]. Although we used hypothetical patients, this did allow us to focus on specific changes in risks across a variety of patients, permitting easy calculation of the relative risk increases due to risk factor change.

## Conclusion

There is considerable variation among CVD risk calculators in the relative risk increase for each specific risk factor. The highest average relative risk increase for a calculator was 3.4–18.2 higher than the lowest average relative risk increase, depending on risk factor. Some calculators more often produce higher relative risk changes (e.g. PROCAM) while others more often produce lower relative risk changes [e.g. Edinburgh (ASSIGN)]. However, there was also similarity among some of the calculators. Although consistency could occur among calculators derived from different databases, 10-year CVD Framingham calculators appeared to have the most consistent relative risk increases. Researchers and clinicians should not assume risk differences from reductions in risk factors are reliable or consistent from one calculator to the next.

## Additional file

**Additional file 1: Table S1.** Comparison of Averages and Ranges of Relative Risk Increases across Risk Factors for Selected Subgroups of Non-diabetic Calculators.

## Abbreviations

CVD: cardiovascular disease
CHD: coronary heart disease
BP: blood pressure
HDL: high density lipoprotein
