# Supplementary material for: Variation among cardiovascular risk calculators in relative risk increases with identical risk factor increases
Source: BMC Res Notes. 2015 Sep 7;8:417. doi: 10.1186/s13104-015-1401-8 (PMC4561470; doi:10.1186/s13104-015-1401-8)
Supplement: Supplementary file 1 — Additional file 1: Table S1. Comparison of Averages and Ranges of Relative Risk Increases across Risk Factors for Selected Subgroups of Non-diabetic Calculators. [file 13104_2015_1401_MOESM1_ESM.doc]

Supplemental Table S1. Comparison of Averages and Ranges of Relative Risk Increases across Risk Factors for Selected Subgroups of Non-diabetic Calculators.

| Comparison of | Sub-Groups (Calculator #) |  | Age  (50 to 70) | Gender  (female to male) | Smoking  (non-smoker to smoker) | Systolic BP (120 to 160 mmHg) | Total Chol  (4 to 7 mmol/L) | HDL  (1.3 to 0.8 mmol/L) |
| --- | --- | --- | --- | --- | --- | --- | --- | --- |
| Outcomes used by Calculators | CVD (9) | Average | 196% | 71% | 64% | 69% | 66% | 45% |
|  |  | Range | 103%-344% | 35%-141% | 37%-87% | 30%-124% | 51%-89% | 27%-60% |
|  | CHD (5) | Average | 213% | 137% | 86% | 49% | 152% | 78% |
|  |  | Range | 82%-395% | 64%-225% | 31%-118% | 16%-72% | 77%-302% | 61%-133% |
| Database used to Derive Calculators | Framingham (4) | Average | 112% | 60% | 64% | 67% | 65% | 48% |
|  |  | Range | 103%-121% | 44%-91% | 59%-70% | 43%-83% | 51%-78% | 41%-56% |
|  | Non-Framingham (5) | Average | 264% | 81% | 65% | 70% | 67% | 43% |
|  |  | Range | 105%-344% | 35%-141% | 37%-87% | 30%-124% | 51%-89% | 27%-60% |
